# Shape-Driven Response of Gold Nanoparticles to X-rays

**DOI:** 10.3390/nano13192719

**Published:** 2023-10-07

**Authors:** Simona Tarantino, Caterina Capomolla, Alessandra Carlà, Livia Giotta, Mariafrancesca Cascione, Chiara Ingrosso, Edoardo Scarpa, Loris Rizzello, Anna Paola Caricato, Rosaria Rinaldi, Valeria De Matteis

**Affiliations:** 1 Department of Mathematics and Physics “E. De Giorgi”, University of Salento, Via Monteroni, 73100 Lecce, Italy; simona.tarantino@unisalento.it (S.T.); mariafrancesca.cascione@unisalento.it (M.C.); annapaola.caricato@le.infn.it (A.P.C.); 2 Oncological Center, “Vito Fazzi” Hospital of Lecce, Piazza Filippo Muratore 1, 73100 Lecce, Italy; capomolla.caterina@gmail.com (C.C.);; 3 Department of Biological and Environmental Sciences and Technologies, University of Salento, 73100 Lecce, Italy; livia.giotta@unisalento.it; 4 Institute for Microelectronics and Microsystems (IMM), National Research Council (CNR), Via Monteroni, 73100 Lecce, Italy; 5 CNR-IPCF S.S. Bari, c/o Department of Chemistry, University of Bari Aldo Moro, Via Orabona 4, 70126 Bari, Italy; c.ingrosso@ba.ipcf.cnr.it; 6 Department of Pharmaceutical Sciences, University of Milan, 20133 Milan, Italy; edoardo.scarpa@unimi.it (E.S.); loris.rizzello@unimi.it (L.R.); 7 The National Institute of Molecular Genetics (INGM), 20133 Milan, Italy; 8 National Institute of Nuclear Physics (INFN), Section of Lecce, Via Monteroni, 73100 Lecce, Italy

**Keywords:** gold nanoparticles, X-rays, radiotherapy, physicochemical properties, medical physics, nanoparticle synthesis

## Abstract

Radiotherapy (RT) involves delivering X-ray beams to the tumor site to trigger DNA damage. In this approach, it is fundamental to preserve healthy cells and to confine the X-ray beam only to the malignant cells. The integration of gold nanoparticles (AuNPs) in the X-ray methodology could be considered a powerful tool to improve the efficacy of RT. Indeed, AuNPs have proven to be excellent allies in contrasting tumor pathology upon RT due to their high photoelectric absorption coefficient and unique physiochemical properties. However, an analysis of their physical and morphological reaction to X-ray exposure is necessary to fully understand the AuNPs’ behavior upon irradiation before treating the cells, since there are currently no studies on the evaluation of potential NP morphological changes upon specific irradiations. In this work, we synthesized two differently shaped AuNPs adopting two different techniques to achieve either spherical or star-shaped AuNPs. The spherical AuNPs were obtained with the Turkevich–Frens method, while the star-shaped AuNPs (AuNSs) involved a seed-mediated approach. We then characterized all AuNPs with Transmission Electron Microscopy (TEM), Uv-Vis spectroscopy, Dynamic Light Scattering (DLS), zeta potential and Fourier Transform Infrared (FTIR) spectroscopy. The next step involved the treatment of AuNPs with two different doses of X-radiation commonly used in RT, namely 1.8 Gy and 2 Gy, respectively. Following the X-rays’ exposure, the AuNPs were further characterized to investigate their possible physicochemical and morphological alterations induced with the X-rays. We found that AuNPs do not undergo any alteration, concluding that they can be safely used in RT treatments. Lastly, the actin rearrangements of THP-1 monocytes treated with AuNPs were also assessed in terms of coherency. This is a key proof to evaluate the possible activation of an immune response, which still represents a big limitation for the clinical translation of NPs.

## 1. Introduction

The use of inorganic nanomaterials in different fields has raised exponentially in recent decades because of their unique physicochemical properties [1]. To this respect, gold nanoparticles (AuNPs) are among the most used nanostructures especially for biomedical applications due to their high biocompatibility caused by their inert chemical nature [2,3].

Several routes based on either bottom-up or top-down approaches have been extensively explored to obtain differently sized or shaped AuNPs [4]. Typically, the bottom-up synthesis is the preferential choice because of its overall feasibility, coupled with reproducibility and a high control over the quality of the final colloidal solution [5].

With regard to radiotherapy, different studies focused on the use of AuNPs as radiosensitive agents, which have been demonstrated to improve the effects of X-rays [6]. This occurs thanks to the Au high atomic number (Z = 79) that triggers a high energy absorption [7]. The result is a high X-ray absorption coefficient of the AuNPs, which results in the emission of secondary electrons after irradiation. In particular, the interaction between NPs and X-rays is explained through the phenomenon of electron excitation and scattering on AuNPs. This phenomenon involves Auger cascades, which are emissions of electrons responsible for the ionization of water molecules [8] and intracellular components.

This increases the radiation dose, which is locally released [9] only in the tumor site, preserving the surrounding healthy tissues from exposure [10].

The radiation enhancement exerted using AuNPs can be influenced with different parameters such as NP size and shape. Therefore, depending on the tumor treatment, it is imperative to use the most suitable shape to maximize the effects of therapy to avoid collateral effects [11]. Several factors must be considered in order to choose a particular NP shape and size [12] for therapeutic efficacy in RT.

The physical principles on the basis of the AuNPs’ radiotherapy enhancement are well known, which can be measured with the dose enhancement factor (DEF), representing the ratio of the radiation dose absorbed by the tumor cells compared with that absorbed without the presence of AuNPs [13]. For example, following Monte Carlo simulations, an increase in the therapeutic dose is demonstrated, and a consequent low radiation dose in cells after uptake of AuNPs [14,15]. Several in vitro and in vivo studies were performed to verify the AuNPs’ efficacy in RT. Teraoka et al. [16] investigated the effect of AuNPs in radiotherapy with an in vitro experiment using a human head and neck carcinoma cell line, HSC-3. The authors showed a strong reduction in tumor volume after 2 Gy, 4 Gy and 8 Gy.

Also, Janic et al. [17] used human triple-negative breast cancer cells (MDA MB 231 TNBC) to investigate how the AuNPs influence in RT using a 15 Gy dose at 160 kV with an X-ray source.

In another experiment, Yoshida et al. [18] created poly(2-methacryloyloxyethyl phosphorylcholine) (PMPC)-modified Au nanocomposites of different shapes (nanospheres, nanorods and NSs) as RT radiosensitizers for breast cancer cell line MCF-7 treatment. Both the therapeutic effect and cellular uptake of PMPC-AuNSs were significantly higher compared to other nanocomposites. This result indicated that the cellular uptake of PMPC-modified Au nanocomposites was influenced by the shape.

Alhussan et al. [19] studied the efficacy of AuNPs in RT creating a co-culture of cancer-associated fibroblasts (CAFs) and pancreatic ductal adenocarcinoma (PDAC) cells, using an MIA PaCa-2 cancer cell line. Co-cultured cells were incubated with AuNPs and then treated with 2 Gy of radiation. Co-cultured tumor cells without RT irradiation showed up to 23% reduction in DNA double-strand breaks (DSBs); on the other hand, AuNPs/RT induced an increase to 25%.

Moreover, there are currently no studies on the evaluation of potential NP morphological changes upon specific irradiations. In this work, we synthesized two different shapes of AuNPs by coupling the standard Turkevich–Frens method with a seed-mediated growth approach, to achieve either spherical or star-like AuNPs, respectively. The NPs were deeply characterized with TEM, UV-vis, FTIR, DLS and zeta potential. Then, we used two different concentrations of NPs (100 µM and 300 µM) for X-ray irradiation experiments in water. A total of 1.8 Gy (which is the most common therapeutic dose in RT) and 2 Gy were used to test the responsiveness of these AuNPs to X-rays. We concluded that the two types of NPs did not change their morphology and physical behavior upon irradiation, making them suitable nanotools for application in medicine. To confirm this last finding, we assessed the impact of AuNSs on THP-1 monocytes, to verify the potential stimulation of an inflammation response in terms of actin rearrangements.

## 2. Materials and Methods

### 2.1. Synthesis of AuNPs

#### 2.1.1. Turkevich–Frens Method

The synthesis of spherical Au NPs was performed following the Turkevich–Frens procedure with some modifications [20]. Briefly, 15 mg of tetrachloroauric (III) acid (HAuCl_4,_ Sigma-Aldrich, Dorset, UK) was dissolved in MilliQ water to obtain a solution having a concentration of 0.25 mM. Then, 150 mL of a HAuCl_4_ solution was moved to a reaction flask and heated at 100 °C until boiling under stirring and reflux. Later, 0.1 M of a sodium citrate (10 mL) aqueous solution was quickly added and a change in a color reaction was observed, which became red wine colored. After cooling to room temperature, the reaction product was stored in the dark in a refrigerated room. Subsequently, the solution was transferred into polypropylene tubes and centrifuged at 7500 rpm for about 45 min followed by a washing step using water. 

#### 2.1.2. Synthesis of AuNSs with “Seed and Growth” Method

The synthesis of AuNSs required two steps:

- Production of the seed solution which was constituted by small AuNPs (5–20 nm) following the protocol described in [21] with some modifications. First, 600 µL of a NaBH_4_ solution (10 mM) was added to a solution of 0.1 mL of HAuCl_4_ (25 mM) and a surfactant, i.e., Triton X (150 mM), under stirring. The mix immediately shifted from light yellow to light red.
- The growth solution was prepared with 20 mL of Triton X (150 mM), 0.4 mL of HAuCl_4_ (25 mM), 0.788 M of ascorbic acid and 100 µL of AgNO_3_ (45 µM) under stirring for 15 min.

Then, a small amount of the seed solution was added to the growth mixture and the solution turned from a yellow to light red/grey color, maintaining the solution under stirring (600 rpm) at room temperature. To obtain the specific nanostar shape, several proofs were performed based on the reaction time. We stopped the reaction after 6 h, 9 h, 12 h and 17 h, showing that the 12 h of the synthetic procedure was the most suitable to obtain star-shaped NPs. After this step, the solution was centrifuged three times with MilliQ water at 3800 rpm for 30 min and then other washes were carried out using a speed of 13,000 rpm to eliminate any reaction residuals.

### 2.2. Characterization of Au-Based Nanomaterials

#### 2.2.1. Transmission Electron Microscopy (TEM)

TEM analyses were performed by using a Jeol Jem-1011 microscope operating at 100 kV, equipped with a high-contrast objective lens and with a tungsten filament as an electron source with an ultimate point resolution of 0.34 nm. Images were acquired using a Quemesa Olympus CCD 11 Mp camera. The samples useful for TEM observations were prepared by depositing a drop (about 10 µL) of the aqueous solution containing the metallic NPs on the carbon-coated grids. Once the water evaporated, the grids were used for the analysis. A size statistical analysis of the NPs’ average size and size distribution was performed by using the fitting analysis available on OriginPro software. SR1 v8.0773.

#### 2.2.2. Uv-Vis and Fourier Transform Infrared (FTIR) Characterization

UV-Vis-NIR absorption spectra of metal NP solutions were performed with a Cary 5000 (Varian) in the range 300–800 nm.

To acquire mid-infrared absorption spectra, a Fourier Transform Infrared (FTIR) spectrophotometer was used (Spectrum One model, Perkin Elmer, Waltham, MA, USA), equipped with a universal attenuated total reflectance (ATR) accessory. The internal reflection element (IRE) was a three-bounce diamond microprism with a 4 mm diameter. Typically, 2 µL of aqueous NPs was cast onto the ATR crystal and the solvent was left to evaporate. Once dried, the ATR-FTIR spectra of samples were acquired at a 4 cm^−1^ resolution. For each spectrum, 16 interferograms were recorded and averaged. The background spectrum was recorded with the bare diamond microprism.

#### 2.2.3. DLS and ζ-Potential

The DLS and ζ-potential acquisitions were recorded with a Zetasizer Nano-ZS having a HeNe laser (4.0 mW) working at a 633 nm detector (ZEN3600, Malvern Instruments Ltd., Malvern, UK) in aqueous solutions (25 °C, pH 7). Size statistical distribution of the NPs was measured on 300 Au NPs fitted with a normal Gaussian function.

#### 2.2.4. Irradiation Set-Up

**Figure 1:**
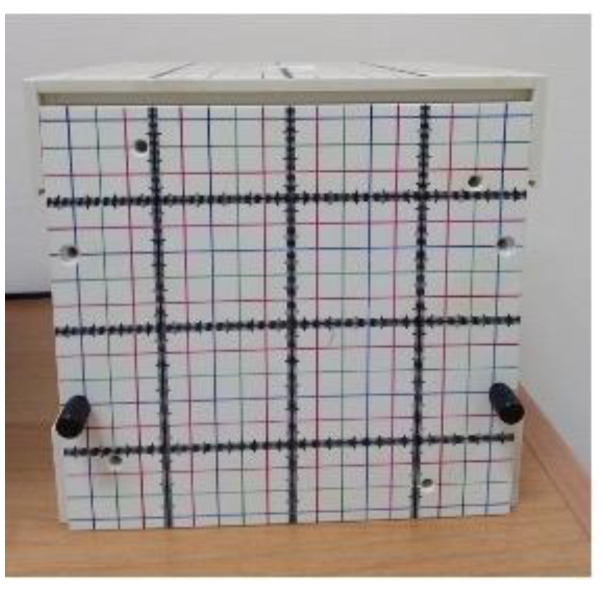
Water-equivalent phantom housing AuNP samples.

AuNP samples were irradiated with a Siemens Primus linear accelerator operating with 6 MV photon beams. AuNP samples were placed in a polystyrene chamber inside a hollow water-equivalent phantom. To achieve a uniform dose distribution, the box was wrapped with a bolus (Figure 1).

The accelerator was calibrated at a distance of the source-ionization chamber of 105 cm and depth of the chamber of 5 cm inside a water-phantom and field of 10 cm × 10 cm under these conditions; 100 Monitor Units (MU) correspond to 100 cGy. MU is the unit of measurement of the dose delivered with the accelerator. In all the other configurations, the MU were calculated considering the field size, the distance to the source and the depth within the body.

### 2.3. X-ray Irradiation

Once the AuNPs were synthesized, two replicates of each sample, i.e., 1 mL of the two shapes of NP solutions having two different concentrations (100 μM and 300 μM), were placed in Eppendorf vials. The X-ray irradiations were performed at the Radiotherapy Department of the ‘Vito Fazzi’ Hospital (Lecce, Italy) using two different doses, i.e., 1.8 Gy and 2 Gy. One sample was placed inside the phantom and a CT scan was performed to set the irradiation treatment plans. For this purpose, four 5 cm × 5 cm beams with angles of 0°, 90°, 180° and 270° were involved. This plan was calculated for the two different prescribed doses with the Treatment Planning System Oncentra. Dose distribution obtained in the planning phase is shown in Figure 1.

**Figure 2:**
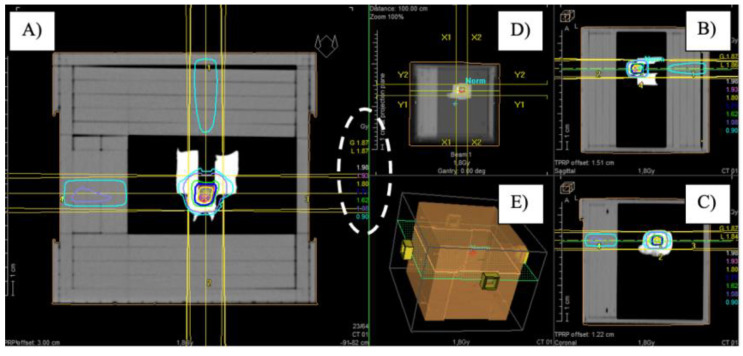
(**A**) Axial, (**B**) sagittal and (**C**) coronal images of the dose distribution in the AuNP sample contained in the phantom; (**D**) view from the accelerator gantry (Beam Eye View—BEV); (**E**) 3D image of the phantom with the irradiation beam inputs. In the white dashed circle was the isodose curves legend.

The irradiation was carried out by delivering the MU specified in the treatment plans, which are reported in Table 1 whereas, a 3D image of the phantom with the irradiation beam input was reported in Figure 2.

### 2.4. THP-1 Culture and Differentiation

Human Leukemic Monocytes (THP-1) (ATCC-TIB-202) were grown in RPMI-1640 with 2 mM l-glutamine, 25 mM HEPES (Sigma-Aldrich, Dorset, UK), 10% (*v*/*v*) fetal bovine serum (FBS, Sigma-Aldrich, Dorset, UK) and 1% (*v*/*v*) penicillin–streptomycin (Sigma-Aldrich, Dorset, UK). THP-1 cells were differentiated into M0 macrophages by using 10 ng/mL of phorbol 12-myristate 13-acetate (PMA, Sigma-Aldrich, Dorset, UK) for 48 h at standard culturing conditions (95% air and 5% CO_2_, at 37 °C).

### 2.5. Confocal Analysis

A Confocal Laser Scanning Microscope (CLSM; Leica SP8, Milton Keynes, UK) was used to acquire images on actin and nuclei. Following the THP-1 differentiation, M0 macrophages were incubated with AuNSs (100 µM and 300 µM) for 24 h and 48 h in an incubator. Then, the cells were washed with PBS (Sigma-Aldrich, Dorset, UK) and fixed with 3.7% formaldehyde (Sigma-Aldrich, Dorset, UK) for 10 min at room temperature. The cell permeabilization step was carried out with Triton X (0.2%) (Sigma-Aldrich, Dorset, UK); then, cells were stained using DAPI (Sigma-Aldrich, Dorset, UK) for nuclei imaging, whereas the CellMask™ Deep Red Plasma Membrane Stain (Thermo Fisher Scientific, Waltham, MA, USA) was used to stain cell membranes.

Coherency of F-actin was performed on 75 cells, using the ImageJ 1.51 software analysis.

## 3. Results and Discussion

### 3.1. Pre-Irradiation Analysis

As of today, the treatment of solid tumors is still based on conventional radiotherapy and chemotherapy that have several side effects due to their low specificity for tumor cells. This means that all cells (pathological and healthy) are reached with chemicals and X-rays upon therapy. In the latter case, the use of nanomaterials based on Au can help to confine the beam only in the tumor site. However, prior to using these materials in biological models, it is necessary to understand if the irradiation with therapeutic doses can perturb their physicochemical properties. Thus, the aim of our work is to verify this.

The Au nanostructures obtained with the two synthetics routes were morphologically characterized with TEM. The TEM analysis was carried out to find out the shape and average size of AuNPs after each synthesis and subsequent purification.

**Figure 3:**
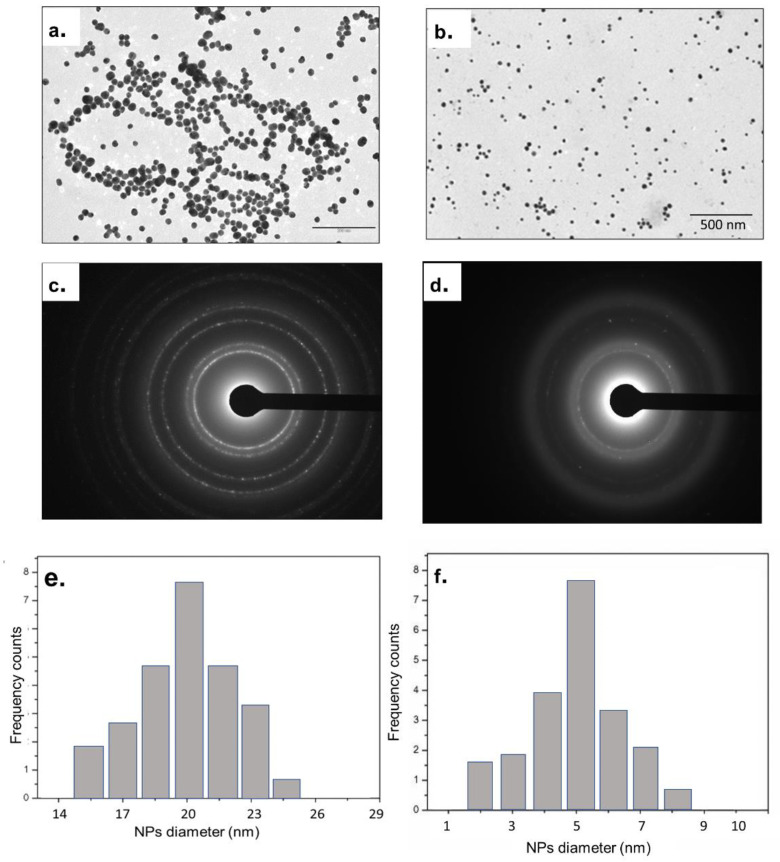
TEM acquisitions of AuNPs obtained by Turkevich–Frens method (**a**), Au seeds (**b**) and the relative SAED patterns (**c**,**d**). Size distribution of Turkevich–Frens AuNPs (**e**) and AuNP seeds obtained with ImageJ software (**f**).

In Figure 3a, we report the TEM image of the AuNPs obtained with the Turkevich–Frens method; NPs were spherical and monodispersed without aggregates, with an average diameter in the range of 20–30 nm (Figure 3a). Selected Area Electron Diffraction (SAED) showed concentric diffraction rings, which reflected the polycrystalline nature of AuNPs (Figure 3c). Ring patterns are typically created when the NP is formed, and the radius of each ring stands for the plane distance [22]. The size distribution analysis assessed with ImageJ confirmed the size of NPs (Figure 3e).

The morphological analysis was also performed on the AuNSs obtained with the “seed and growth” approach. Compared with the in situ synthesis, this method is based on a step-by-step procedure able to have a great control of nano-object shape and size.

First, since the synthetic method requires the production of seeds as nucleation centers to grow the Au branches, the TEM analysis was conducted on them to verify the size, the monodispersion and the purity of the colloidal solution (Figure 3b). The Au seeds were spherical in shape, polycrystalline (Figure 3d) and they exhibited a small size around 5 ± 4 nm (Figure 3f).

Then, the growth step involved the reduction of HAuCl_4_ on Au seeds with ascorbic acid and a polymer or surfactant; in our case, Triton X100 was used as a capping agent.

The latter agent was preferentially absorbed on specific crystalline facets of the Au seeds, inducing the growth step along specific crystallographic directions [23]. In addition, AgNO_3_ was added to enhance the shape control and the yield of branches [24,25].

In order to obtain uniform and multibranched AuNSs, the growth reaction was stopped at different time points (6, 9, 12 and 17 h) and the morphological evolution of the AuNSs was evaluated with TEM.

**Figure 4:**
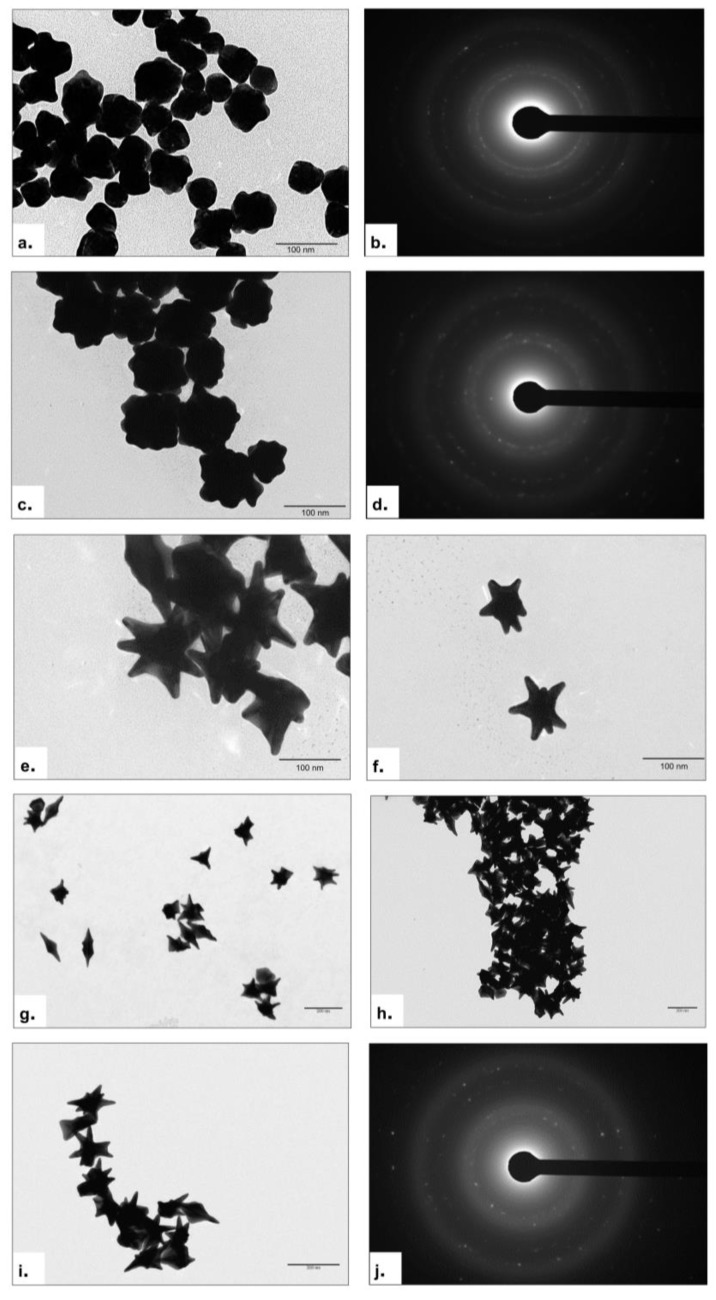
TEM acquisitions of the AuNSs after 6 and 9 h of synthetic approach (**a**,**c**) and relative SAED pattern (**b**,**d**). TEM images of AuNSs obtained after 12 h of reaction time at different magnifications (**e**–**i**). SAED pattern of AuNSs (**j**).

After 6 h of reaction, the nano-objects obtained showed a size of around 60 nm with a morphology slightly squared off, with small prominences on the surface (Figure 4a). The polycrystallinity of NPs is shown in SAED images (Figure 4b). After 9 h of reaction time, the morphology changed towards the presence of slightly more acuminate tips on the surface of the AuNSs, together with an increase in size (60–100 nm) (Figure 4c). Figure 4d shows the respective SAED pattern with a bit larger concentric rings due to the increase in the core and spikes’ dimension. After 12 h of reaction time, the desired shape was obtained. As shown in Figure 4e–i, the star shape was entirely formed, exhibiting a spherical core-structure with long spikes on the surface. The branches’ size did not exceed 40 nm, while the total size of the AuNSs was measured to be around 70 nm. Also, the SAED pattern confirmed the complete growth due to the presence of concentric rings significantly larger than the previous ones (Figure 4j).

TEM investigation allowed a preliminary morphology study of AuNSs obtained with the seed and growth method. The TEM measurements demonstrated that the reaction time was crucial for obtaining the star shape. After 6 hours, acuminate apexes begin to emerge from the surface of the NP, reaching a maximum growth after 12 h. If the reaction time was extended beyond 12 h, the AuNSs lose their morphology. Indeed, after 17 h, AuNSs appeared to be significantly deformed, without a visible star shape, with spikes of several sizes. In addition, the NPs were aggregated with a high colloidal instability.

**Figure 5:**
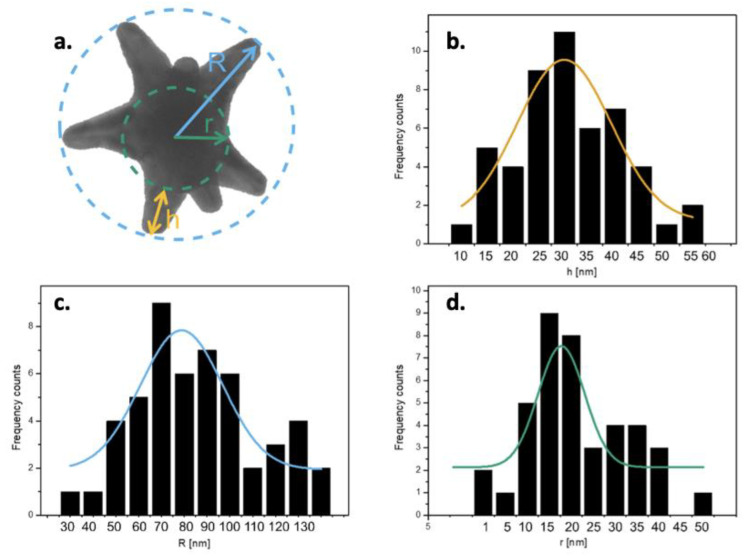
Representative image of the dimensional analysis of three AuNS components, i.e., the length h of the branches, the core-tip distance R and the core diameter r (**a**); size distributions of the components h, R and r, (**b**–**d**), respectively, performed with ImageJ software.

In Figure 5, a morphometric analysis of the size of three AuNS components is presented, namely the branch length (h), the core-tip distance (R) and the core diameter (r).

In Figure 5b–d, the size distribution of each of the three components (h, R and r, respectively) of the population of AuNSs is reported, analyzed with ImageJ software. On each of the three size distributions, the relative Gaussian curve is represented, pigeonholed on the most prevalent value of h, R and r. Specifically, h averaged 29.4 ± 4 nm (Figure 5b), R averaged 78.2 ± 3 nm (Figure 5c) and r averaged 17.5 ± 2 nm (Figure 5d). We moved on to analyze the surface charge with zeta potential (ZP) measurements.

Table 2 reports the ZP values of the Au nanostructures with their standard deviations. All AuNPs exhibited negative ZP values and the highest ZP value was found for the Turkevich–Frens AuNPs with a ZP value of −20 ± 2 mV. These data indicated that the solution was characterized by high stability, preventing any unintended aggregation. Regarding seeds and AuNSs, the values of the ZP were lower than those of the other AuNPs. In detail, the seeds presented a ZP value of −11 ± 1.3 mV; the AuNSs obtained with reaction times of 6, 9 and 12 h exhibited ZP values of −16 ± 3, −20 ± 2 and −24 ± 2 mV, respectively.

To confirm the data obtained with the TEM analysis, we moved on to measure NP solutions with Dynamic Light Scattering (DLS), a technique used for the determination of both size and size distribution of particles [26,27]. The third column of Table 2 shows the values of the hydrodynamic diameter with their standard deviations for each type of AuNP. For the Turkevich–Frens AuNPs, the hydrodynamic diameter was 19 ± 2 nm, slightly more than the average of the size values extracted from the TEM analysis. Also, both seeds and AuNSs revealed a marginally larger hydrodynamic diameter compared to the size calculated with TEM. In fact, more specifically, the seeds exhibited a hydrodynamic diameter of 7 ± 5 nm; the AuNSs with a reaction time of 6, 9 and 12 h exhibited a hydrodynamic diameter of 60 ± 6, 68 ± 3 and 70 ± 2 nm, respectively.

Uv-vis spectroscopy provides information about the plasmonic absorption of AuNPs from which their average diameter can be estimated. It happens because differently sized AuNPs exhibit unique light-scattering properties [28]. Generally, the plasmonic resonance peak at 520 nm is related to AuNPs with a very small size and a most likely spherical shape.

**Figure 6:**
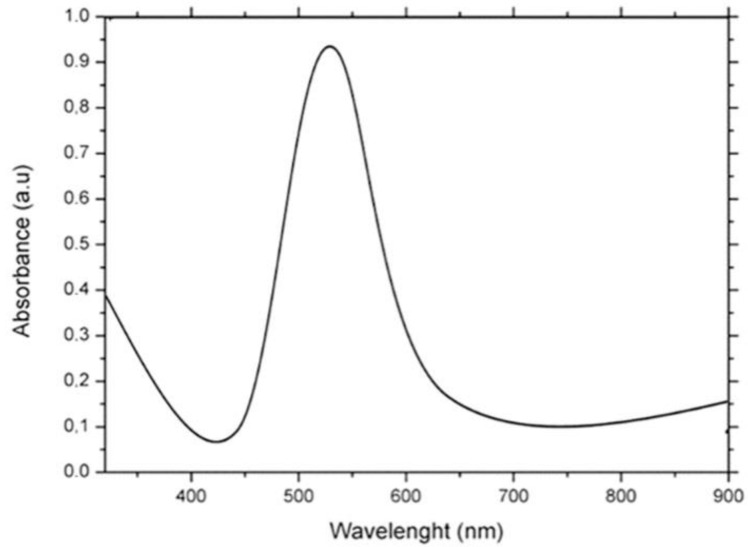
Uv-vis spectra of AuNPs obtained with Turkevich–Frens method.

Figure 6 showed the UV-vis spectrum of Turkevich–Frens AuNPs. A single absorption peak at 520 nm is visible with a high absorbance level [29]. As it turned out from the TEM images, these AuNPs were spherical in shape and around 20 nm in size.

**Figure 7:**
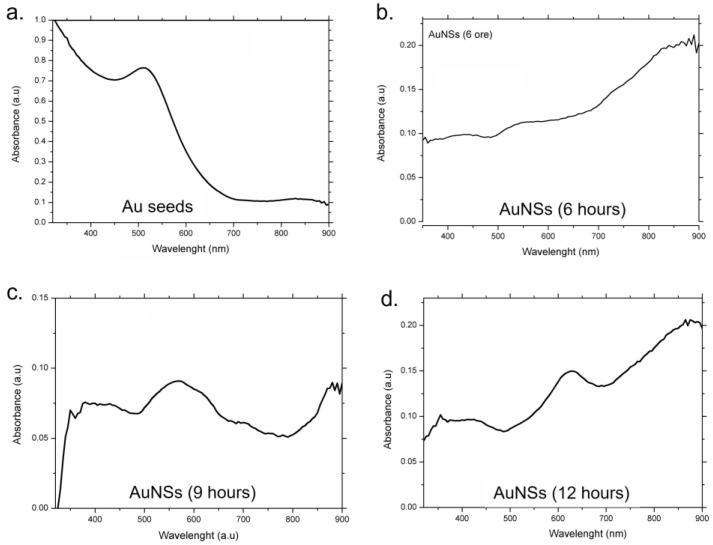
UV-vis spectra of Au seeds (**a**) and AuNSs after 6 h (**b**), 9 h (**c**) and 12 h (**d**) of reaction.

Then, careful UV-vis measurements of AuNSs were performed, starting from seeds (Figure 7). In Figure 7a, the spectrum acquired on Au seeds is presented; the NPs show only a plasmon peak at 520 nm.

In Figure 7b–d, plasmon spectra of AuNSs at different time points of the reaction are reported. After 6 h of synthesis time (Figure 7b), the AuNSs did not exhibit absorption in the visible region, but only in the Near-Infrared Region (NIR), with a redshift visible as peaks from 870 nm to 900 nm, due to the initial formation of the tips on the NPs’ surface [30]. At the intermediate reaction time (9 h), the AuNSs displayed a wide peak in the visible region (500 to 650 nm) with relatively low absorbance levels compared to the seeds, and very narrow peaks in the NIR (Figure 7c).

The former absorption band, which is usually much wider than the 520 nm peaked one, should be produced with the plasmon–plasmon interaction among the aggregated anisotropic AuNPs. It is often referred to as a shoulder rather than a peak, dependent on the degree of the aggregation morphology. After 12 h of reaction time, with the complete formation of the AuNSs, the absorbance level increased, and led to the formation of very narrow peaks in the NIR range (860–900 nm) together with a peak in the visible band between 610 and 640 nm (Figure 7d).

### 3.2. Post-Irradiation Analysis

Following the initial characterization of the nanomaterials, samples were first irradiated with an X-ray beam according to the protocol described in the Section 2 and then they were characterized to evaluate any morphological alterations and/or changes in their properties after X-ray irradiation.

**Figure 8:**
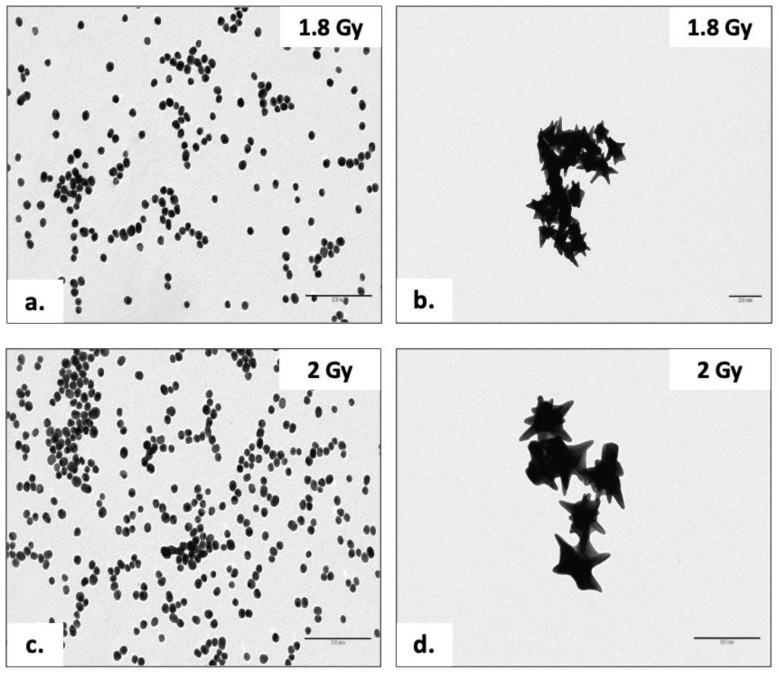
TEM acquisitions of the Turkevich–Frens AuNPs (**a**) and AuNSs (**b**) after 1.8 Gy irradiation dose; TEM acquisitions of the Turkevich–Frens AuNPs (**c**) and AuNSs (**d**) after 2 Gy irradiation dose.

Figure 8 shows the TEM acquisitions of the AuNPs after irradiation with the two radiation doses at 1.8 and 2 Gy. In detail, Figure 8a,b represented Turkevich–Frens AuNPs and AuNSs, respectively, after 1.8 Gy irradiation. In contrast, Figure 8c,d depicted the same duplicate AuNP samples after 2 Gy irradiation. No alteration in the morphology was detected. In fact, all AuNPs exhibited the same shape and size as non-irradiated AuNPs. The perfect spherical shape of the Turkevich–Frens AuNPs continued to persist, along with their relative size of between 20 and 30 nm (Figure 8a,c). Finally, the AuNSs (Figure 8b,d) displayed a total dimension of no more than 100 nm and a count of spikes per NP of six, with a single dimension that did not exceed 50 nm.

The two doses did not even affect the dispersion of the AuNPs. They did not exhibit any kind of aggregation, allowing an initial positive assessment regarding the solution stability.

**Figure 9:**
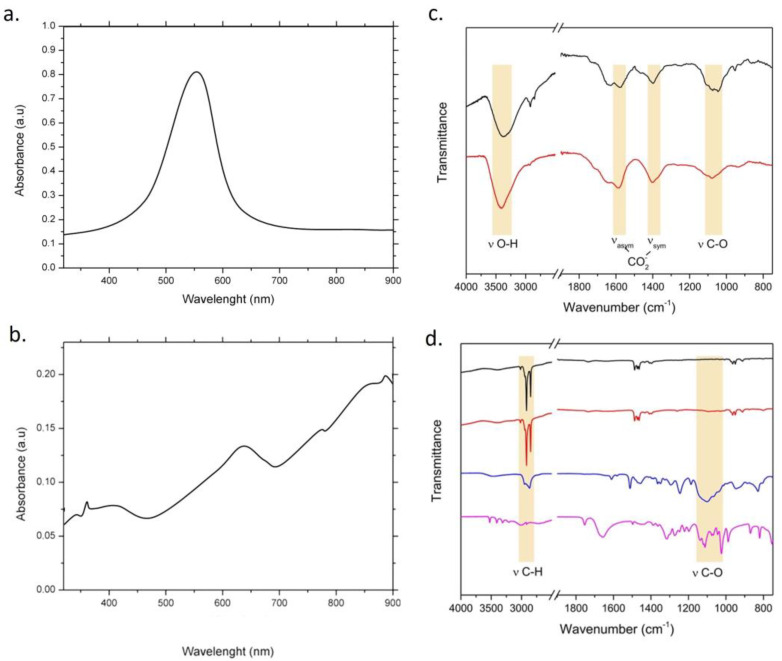
UV-vis absorption spectra of AuNPs achieved with Turkevich–Frens method (**a**) and AuNSs from seed and growth approach (**b**) after 1.8 Gy X-ray radiation. ATR-FTIR spectra of Turkevich–Frens AuNPs before (black trace) and after (red trace) X-ray irradiation (1.8 Gy) (**c**) and ATR-FTIR spectra of AuNS samples and growth organic components (**d**). From top to bottom of (**d**): non-irradiated AuNSs (black trace), AuNSs after exposure to 1.8 Gy X-ray radiation (red trace), Triton X-100 (blue trace) and ascorbic acid (purple trace). ATR-FTIR spectra were suitably normalized and stacked to enable the easy comparison of qualitative features. The yellow panels in (**c**,**d**) outline the spectral regions where the absorption bands due to stretching (ν) vibration modes of specific functional groups can be found.

Both UV-vis and ATR-FTIR spectra of AuNPs after 1.8 Gy irradiation were shown in Figure 9. In particular, Figure 9a,c represented the UV-vis spectra of irradiated Turkevich–Frens AuNPs and irradiated AuNSs, respectively. Figure 9b,d depict the ATR-FTIR spectra of the same irradiated samples, respectively. As observed in the pre-irradiation phase, the UV-vis spectrum of the Turkevich–Frens AuNPs after the exposure phase similarly presents a single peak in the visible range, between 540 and 560 nm (Figure 9a). Likewise, the UV-vis spectrum of the irradiated AuNSs did not differ from that of the non-irradiated AuNSs. In detail, a peak emerged in the visible range (between 640 and 660 nm) and a peak in the NIR band (at about 990 nm), with increasing absorbance levels (Figure 9b).

The Fourier Transform Infrared (FTIR) spectroscopic analysis was performed both on non-irradiated and on irradiated AuNP samples, to evaluate chemical compounds on the AuNPs’ surface. The FTIR spectrum of a specific unirradiated sample was paired with the corresponding FTIR spectrum of the same irradiated sample. Moreover, the reported FTIR spectra of irradiated AuNPs were those in which the 1.8 Gy radiation dose was used, leaving out the 2 Gy dose. This was because it was noticed that there was no spectral distinction between the AuNPs irradiated with the two doses. The ATR-FTIR spectra of Turkevich–Frens AuNPs (Figure 9c, black trace) were dominated with broad signals ascribable to the citrate ligand, in agreement with the well-known coordinating abilities of this compound. Specifically, peaks at 1586 cm^−1^ and 1401 cm^−1^ could be assigned to asymmetric and symmetric stretching vibrations of carboxylate groups [31], while the intense absorption band at 3404 cm^−1^ arose from the stretching mode of hydroxyl moieties [32]. Further signals in the 1200–1000 cm^−1^ range could be attributed to the stretching vibration of alcoholic C-O bonds [32]. X-ray irradiation did not modify the infrared absorption pattern, arising from comparison of spectra acquired on non-irradiated (black trace) and irradiated (red trace) AuNPs. The stability of the citrate capping shell of Au NPs in the experimental conditions employed was thus demonstrated.

In Figure 9d, the FTIR spectra of AuNSs and growth organic components are reported. The AuNSs’ IR spectra, acquired before (black trace) and after (red trace) X-ray irradiation, presented very similar absorption patterns, suggesting that the X-ray exposure did not induce any significant change in the chemical composition of the original sample, at least in terms of the infrared light-absorbing component. The mid-infrared features may be referred mainly to the organic molecules retained in the preparation after the multiple washing steps of AuNSs. At first glance, the IR absorption bands arising from our NS preparation appeared to be quite different than those produced with the organic compounds added in the growth solution, namely Triton X-100 and ascorbic acid (AA), whose standard spectra are reported in the same figure for comparison (blue and purple traces, respectively). Although the oxidized form of AA, which was produced in the growth solution, should present a slightly different absorption pattern, the fact that the main features of reduced AA (purple trace) could not be identified in NS spectra revealed that this small soluble molecule acted only as a reductant in the growth solution, with a scarce ability to establish interactions with AuNSs’ surface. On the other hand, it is well known that Triton X-100 could adhere to the (111) Au faces during crystal growth [33], playing a role in stabilizing and directing NS synthesis. However, unlike ionic detergents, non-ionic surfactants bind weakly to metal NPs [33]. The multiple washing steps performed are thus expected to result in detergent-free preparations. Therefore, the absence in AuNSs’ spectrum of Triton X-100 marker bands, such as the typical signals in the 1200–1000 cm^−1^ range ascribable to the C-O-C asymmetric stretching vibrations of the polyoxyethylene chain [34], would suggest the efficient removal of the detergent in the AuNS sample. In this context, the most intense signals appearing in ATR-FTIR spectra of AuNSs at 2960 cm^−1^ and 2872 cm^−1^, typically due to asymmetric and symmetric stretching modes of methylene groups [32], could be ascribed to some contaminant species bearing aliphatic chain functionalities. The possibility to detect the signals of a very small amount of contaminant species in NP preparations is not surprising, given the ability of metal NPs to enhance the infrared absorption of surrounding molecular layers, in agreement with the well-known SEIRA (Surface Enhanced Infrared Absorption) phenomenon. Hence, ATR-FTIR AuNSs’ spectra might indicate that a small amount of adventitious organic compounds, other than AA and Triton X-100, were present in the final preparation and they were either strictly associated with AuNSs or simply coprecipitated with the NPs during the centrifugation steps. However, the attribution of infrared signals of AuNSs’ infrared spectra to tightly bound Triton X-100 molecules cannot be excluded, considering that the specific interactions and conformation of adsorbed surfactant molecules onto inorganic surfaces may result in a quite different IR absorption pattern with respect to the pure compound in the solid or melted state [35].

In addition to morphological and spectroscopic assessment, we moved to acquire the DLS and zeta potential measurements after irradiation to 1.8 Gy and 2 Gy. As reported in Table 3, no relievable differences were observed with respect to the pre-irradiation analysis.

Considering that AuNSs showed promising chemical–physical properties, and in the perspective of a potential application in vitro in combination with radiotherapy, we assessed their toxicity on THP-1 cells, a monocyte cell line particularly suitable for assessing immunological responses. In particular, we evaluated any possible alteration in the actin rearrangement following administration of 100 or 300 µM of AuNSs for 24 or 48 h.

**Figure 10:**
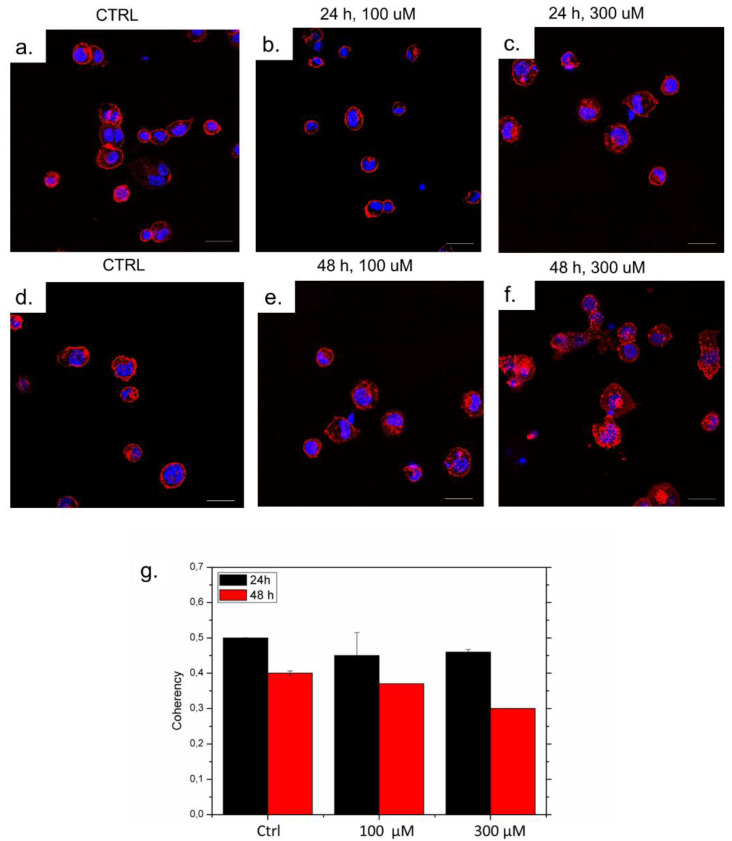
(**a**–**f**) Confocal acquisitions of THP-1 cells treated with 100 uM and 300 uM of AuNSs for 24 h and 48 h. Coherency values applied to confocal acquisitions with ImageJ software (**g**).

Confocal imaging showed a slight actin modification after a 48 h incubation with the highest concentration of AuNSs (Figure 10). Indeed, the fluorescence signal relative to actin in the treated cells was only slightly more disperse and disorganized compared to the untreated control cells (Figure 10a,d). This indicated that AuNSs were well tolerated by the cells. Then, we analyzed the coherency value (Figure 10g) that represented the actin organization. The value ranges from 0 to 1, with 1 indicating highly oriented structures and 0 indicating isotropic areas [36]. The analysis confirmed that only at high concentrations of AuNSs for 48 h, the actin started to be disorganized. Then, the low concentration of these nanostructures resulted in more appropriateness for future application in vitro and in vivo in cancer cells.

## 4. Conclusions

Spherical and star-shaped AuNPs were synthesized by means of the standard Turkevich–Frens method and seed-mediated growth, respectively. The aim of the work was to check whether two X-ray irradiation doses (1.8 Gy and 2 Gy) could trigger alterations in the morphology and physicochemical properties of both AuNPs. In particular, the 1.8 Gy is the typical radiation dose delivered to patients during radiotherapy treatment. The AuNPs were analyzed both before and after irradiation, with TEM, UV-vis, FTIR characterization DLS and zeta potential analyses. No modifications were revealed in the AuNPs exposed to the radiation doses. In addition, since the AuNSs showed enhanced plasmonic properties compared to spherical NPs, their stimulation of immune cells was analyzed on human macrophages, with the aim of exploring potential clinical applications. The results achieved revealed that only high doses of AuNSs for a prolonged time can alter the actin reorganization of cells.

The data obtained support the hypothesis that AuNSs can be used in radiotherapy without altering either their properties or the surrounding biological environment, thus being able to guarantee excellent results.

## Figures and Tables

**Table 1:** Values of the MU delivered with the accelerator for each gantry angle in the required prescriptions.

	Angles	0°	180°	90°	270°
Prescription					
1.8 Gy		74 MU	37 MU	61 MU	63 MU
2 Gy		83 MU	41 MU	68 MU	71 MU

**Table 2:** ZP values with respective standard deviations and the hydrodynamic diameter values obtained with the DLS analysis ± SD.

AuNP Type	Zeta Potential ± SD (mV)	Hydrodynamic Diameter ± SD (nm)
Turkevich–Frens	−20 ± 2	19 ± 2
Seeds	−11 ± 1.3	7 ± 5
Stars (6 h)	−16 ± 3	60 ± 6
Stars (9 h)	−20 ± 2	68 ± 3
Stars (12 h)	−24 ± 2	70 ± 2

**Table 3:** DLS and zeta potential acquired on AuNPs and AuNSs in water after 1.8 Gy and 2 Gy X-ray irradiation.

AuNP Type (Irradiation, 1.8 Gy)	Zeta Potential ± SD (mV)	Hydrodynamic Diameter ± SD (nm)
Turkevich–Frens	−21 ± 3	20 ± 1
Stars	−27 ± 2	72 ± 1
AuNP Type (Irradiation, 2 Gy)	Zeta Potential ± SD (mV)	Hydrodynamic Diameter ± SD (nm)
Turkevich–Frens	−19 ± 4	19 ± 1
Stars	−22 ± 3	68 ± 3

## Data Availability

The data presented in this study are available in this article.
